# Disordered Eating and Service Contact in a Representative UK Sample

**DOI:** 10.1192/bjo.2024.147

**Published:** 2024-08-01

**Authors:** Benjamin Geers, Helen Bould, Tamsin Newlove-Delgado

**Affiliations:** 1 Children and Young People's Mental Health Research Collaboration (ChYMe), University of Exeter Medical School, Exeter, United Kingdom; 2 Centre for Academic Mental Health, Bristol Medical School, University of Bristol, Bristol, United Kingdom; 3 Gloucestershire Health and Care NHS Foundation Trust, Gloucester, United Kingdom

## Abstract

**Aims:**

Aim 1: Identify the proportion of 11–19-year-olds in the Mental Health of Children and Young People 2017 survey screening positive for a possible eating problem, using the Development and Wellbeing Assessment.

Aim 2: Describe patterns of service contact in individuals screening positive for possible eating problems.

**Methods:**

We used data from the Mental Health of Children and Young People 2017 survey, a stratified probability sample of children and their parents and teachers across England. The screening questions from the Eating Disorders module of the Development and Wellbeing Assessment (DAWBA) was completed by all children aged 11–19, all parents of children ages 11–16 and parents of children ages 17–19 where consent was given. Individuals were classified as screening positive for possible eating problems if they had one or more self-reported symptoms, or two or more parent-reported symptoms. We describe the proportion of individuals screening positive by age, sex, co-morbidities, and household income.

Individuals also answered questions about help seeking from different sources (in relation to any mental health concern). We classify these sources of support as informal, professional and specialist.

We analysed all data using Stata 17.

**Results:**

A total of 36.4% (95% CI 34.8, 38.1) of children and young people aged 11–17 in England screened positive for a possible eating problem, including 47.6% of females (95% CI 45.3, 50.0) and 25.6% of males (95% CI 23.7, 27.8). 60.7% (95% CI 57.9, 63.4) of individuals who screened positive reported that they received no help over the previous year; 13.1% (95% CI 11.2, 15.4) had received informal help only; 17.0% (95% CI 15.0, 19.3) had professional but not specialist help; and 9.13% (95% CI 7.67, 10.9) had received specialist help. High proportions of individuals who received support from formal services during the year prior to the survey screened positive for possible eating problems: 42.7% (95% CI 38.6, 47.0) for teachers, 46.9% (95% CI 40.9, 53.0) for GPs, 32.2% (95% CI 23.1, 42.9) for Paediatrics and Child Health and 50.0% (95% CI 43.1, 56.8) for Mental Health Services.

**Conclusion:**

Despite high numbers of young people screening positive for a possible eating problem, rates of help seeking in this group were low. Conversely, high proportions of those seeking professional help have a possible eating problem.

Clinicians should be aware of the high proportions of individuals with possible eating problems accessing their services. Future research should aim to increase help seeking in individuals with possible eating problems.

